# SOCIUS Mentoring—A Novel Course to Encourage Students for a Career as Surgical Oncologists

**DOI:** 10.3390/medsci10030035

**Published:** 2022-06-24

**Authors:** Rüdiger Klapdor, Moritz Kleine, Tobias Schilling, Stephan Huusmann, Anja Philippeit, Jill Philippeit, Kai Timrott, Marcus Kruppa, Peter Hillemanns, Florian Imkamp

**Affiliations:** 1 Department of Obstetrics and Gynecology, Hannover Medical School, 30625 Hannover, Germany; anja.philippeit@gmail.com (A.P.); philippeit.jill-caren@mh-hannover.de (J.P.); kruppa.marcus@mh-hannover.de (M.K.); hillemanns.peter@mh-hannover.de (P.H.); 2 Department of General, Visceral and Transplant Surgery, Hannover Medical School, 30625 Hannover, Germany; moritz.kleine@vinzenzkrankenhaus.de (M.K.); timrott.kai@mh-hannover.de (K.T.); 3 Clinic of General and Visceral Surgery, Vinzenzkrankenhaus Hannover, 30559 Hannover, Germany; 4 Department of Cardiac, Thoracic, Transplantation and Vascular Surgery, Hannover Medical School, 30625 Hannover, Germany; schilling.tobias@mh-hannover.de; 5 Department of Urology and Urological Oncology, Hannover Medical School, 30625 Hannover, Germany; huusmann.stephan@mh-hannover.de (S.H.); florian.imkamp@vinzenzkrankenhaus.de (F.I.); 6 Urology Clinic, Vinzenzkrankenhaus Hannover, 30559 Hannover, Germany

**Keywords:** gynecologic oncology, surgical oncology, mentoring, surgical education, shortage of doctors, skill training

## Abstract

Surgical disciplines are affected by an increasing shortage of young doctors. Studies show that formerly interested students decide against a career in surgical disciplines at the end of their studies or during practical year. Measures to counteract this development are urgently needed. As a joint project between gynecology, urology, and general surgery, SOCIUS mentoring was designed to prepare and encourage students for a career in surgical oncology. The structured curriculum of SOCIUS mentoring contains six modules, including surgical skills, soft skills, mentoring, theory, clinical visitation, and congress participation and runs over one year. Effects on confidence towards physician skills and plans for a future career were evaluated with questionnaires. After participation, students reported increased confidence in surgical and soft skills. In addition, participants noted that they have specified their career goals and gained more confidence in surgery, as well as seeing more development potential for a career in surgery. We describe the implementation of a novel extracurricular program for motivated students that combines individual mentoring with surgical and soft skills training. Due to its modular structure, this concept can easily be transferred to other disciplines. SOCIUS mentoring, with its combination of mentoring and skills training, is a promising measure to prepare and motivate students for their surgical career and thus counteract the shortage of young talent.

## 1. Introduction

The shortage of physicians is a growing challenge in all fields of medicine [1]. Surgical disciplines in particular suffer from a lack of young talent. The majority of students formerly interested in surgery decide against a surgical career during the practical year or their first years as residents [2]. Limited earning opportunities, a high workload, and a poor work-life balance are causing many young physicians to work in professional fields beyond direct patient care. According to the most recent survey on students in their practical year (final year of medical studies in Germany), students prior to the surgical term indicated that their motivation to work as a physician was 7.4 on a scale of 1–10. After completing the term, this motivation decreased to 5.5 out of 10 points [3]. In order to ensure high-quality medical care in the future a systematic solution to counteract the shortage of young physicians is required [4].

Therefore, students should be encouraged for a career in academic surgery during their undergraduate years. Courses that introduce students to hands-on surgical skills have been shown to have positive outcomes on surgical skills and confidences [5]. Overall, study results suggest that students’ interest in surgical careers increase when they are supported early in the acquisition of surgical skills [6]. Despite proven positive effects on students’ research interest and career planning, systematic mentoring as well as skills training is only sporadically established in various medical fields [4,7,8,9,10,11,12].

Curricula that combine mentoring and surgical skills training have not previously been described in the form presented in this paper. Combining the two measures could lead to increased motivation of students to pursue surgical careers. Therefore, the departments of Gynecology, Visceral Surgery, and Urology at the Hannover Medical School have launched the SOCIUS mentoring program (Surgical Oncology Curriculum for Individual support of ambitious Students). The aim of this program is to accompany motivated students on their way into surgical oncology training providing a curriculum optimally tailored to teach the skills required for the start of an academic career in surgery. Here, we describe the structure of the program and the results after two years.

## 2. Materials and Methods

Students were informed about the program via an email, posters, and short presentations during curricular lectures. Students between their fifth and twelfth semesters (4th–5th year of medical studies) were eligible to apply. Students were selected in a two-stage process. Each participating department evaluated a one-page motivation letter and the curriculum vitae and selected 10 students. These students were then invited for an interview. Selection was based on interest and motivation for surgical oncology, research, and university career. The number of participants was limited to 10 students per year.

**Figure 1:**
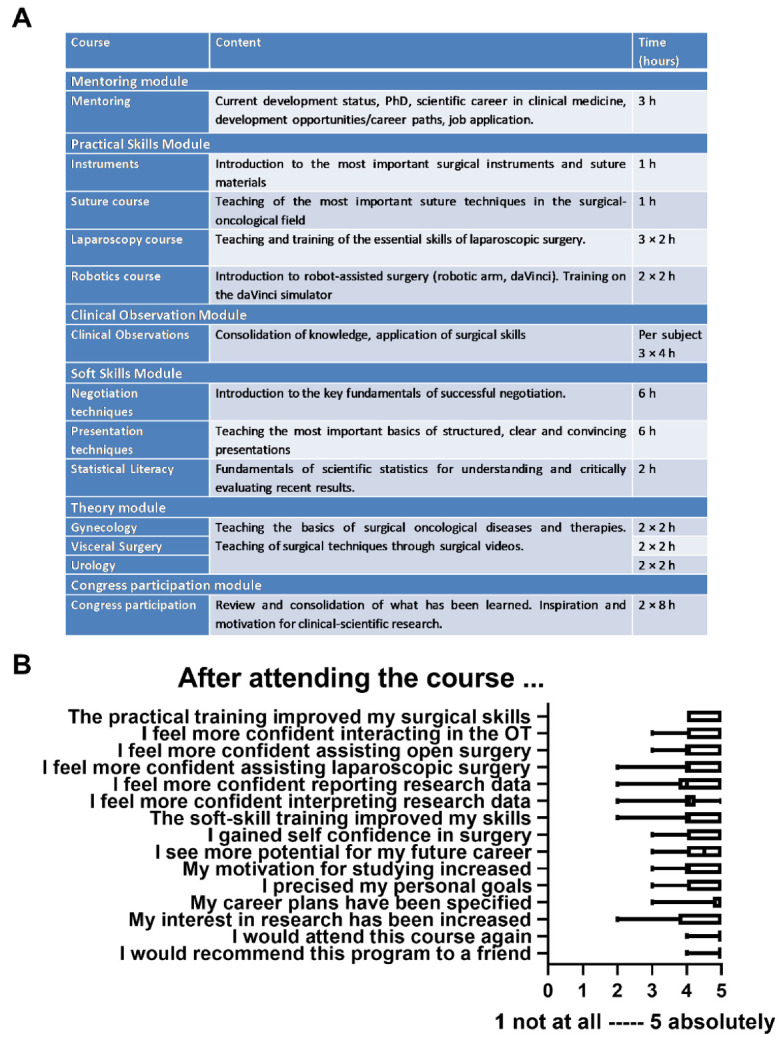
(**A**) The SOCIUS Mentoring Program. Structure and content of the curriculum. (**B**) Boxplots showing the evaluation of the course by the students after completion of the modules (1 bring the lowest agreement and 5 being the highest agreement).

The curriculum included approximately 100 hours of mentoring and training. An attendance rate of 80% was required for successful participation. The curriculum consisted of six modules, which are shown in Figure 1A. The central element was individual mentoring by an experienced senior physician from one of the three participating departments. Over the entire program period, three individual meetings occurred with predefined discussion points. In terms of content, the focus was on professional and personal development, the specification of personal life, and career goals.

After a general introduction to the rules and procedures in the operating room, the “Practical Skills” module included practical seminars on surgical instruments, a suturing course, and intensive laparoscopic surgical training on a virtual laparoscopy simulator LAP Mentor III (Simbionix Ltd., Lod, Israel). Furthermore, functionality of the Da-Vinci surgical robot is explained and basic skills are taught on the DaVinci-Xi simulator (Intuitive Surgical, Sunnyvale, CA, USA).

The “Soft Skills” module included professional training in presentation and negotiation techniques, as well as courses on statistical literacy. In this module, participants were first introduced to established communication techniques that were not routinely taught in healthcare education. Respectful communication with all stakeholders in the hospital, effective patient presentation, presentation of scientific work, and career-enhancing self-marketing were just as much a part of the courses as asserting one’s own interests with the help of comprehensive negotiation techniques.

In each of the three disciplines, there were individual clinical observations with the participating mentors to consolidate and apply what has been learned in clinical settings. Optionally, all participants had the opportunity to attend a national congress.

Before and after participation in the SOCIUS program, participants’ confidence was evaluated using a Likert-scale questionnaire, which was based on evaluations of similar programs and had been validated by experts [10,13]. Furthermore, laparoscopic skills were assessed before and after training using standardized exercises on the LAP Mentor III. Statistical analysis were performed using Microsoft Excel 2010 (Microsoft Corp., Redmond, WA, USA), IBM SPSS version 24 (IBM Corp., Armonk, NY, USA), and Graphpad Prism 8 (Graphpad Software, San Diego, CA, USA). The Wilcoxon matched pairs signed rank test was used to compare continuous variables. The distribution of categorical variables was evaluated by chi-square test. A *p* value <0.05 was considered significant.

All participants gave written consent to the scientific evaluation of the program. The data were stored in pseudonymized form. A positive ethics vote (No. 8806_BO_K_2019) by the ethics committee of the Hannover medical school was available. All methods were carried out in accordance with relevant guidelines and regulations. The implementation of the program was funded by the Dräger Foundation. The LAP Mentor III was funded by the support of the Rudolf Bartling Foundation.

## 3. Results

### 3.1. Course of the Program

A total of 96 students applied to participate in the program in 2019 and 2020. Of these, 20 (20.8%) students were accepted into the program after a multi-stage selection process. The number of female participants was 80%, which represented the general population of medical students in Germany. The baseline characteristics of the participants are shown in Table 1.

During the peak phases of the COVID-19 pandemic, three seminars were conducted as webinars. Several seminars were held in hybrid format offering live streaming. The option for congress participation was taken by 13 (65%) of the students by attending the congress of the German Society of Gynecology and Obstetrics 2020 in Munich and the congress of the European Society of Gynaecological Oncology in Prague. Other planned congress attendances were cancelled due to the COVID-19 pandemic.

### 3.2. Evaluation of the Program

A total of 17/20 (85%) participants participated in the evaluation for the program. Figure 1 shows high to very high satisfaction regarding program goals among SOCIUS mentoring participants. Specifically, students confirmed significant improvement in surgical skills (MW 4.6, SD 0.50) and surgical confidence (MW 4.5, SD 0.61). The lowest, but still positive, rating was given to confidence for reporting statistical results (MW 4.0, SD 0.84).

An important goal of the mentoring was to support the students in their future career planning. Thus, high to very high agreement values were given for the items “potential for future career” (MW 4.4, SD 0.62), “specification of personal goals” (MW 4.6, SD 0.59), and “specification of career plans” (MW 4.7, SD 0.69). Overall mentoring was rated with the highest score (MW 4.9, SD 0.24) and the highest possible value regarding the recommendation of the program to friends (MW 4.9, SD 0.24), as illustrated in Figure 1.

### 3.3. Development of Laparoscopic Skills

The total time required to complete standard laparoscopic exercises on LAP Mentor III was significantly reduced in four out of five tasks after participation in the training (see Supplementary Figure S1). In particular, in exercise 9, which had the highest level of difficulty, participants achieved a reduction in mean total time from 341 s to 252 s (*p* = 0.017).

### 3.4. Confidence of the Students

**Figure 2:**
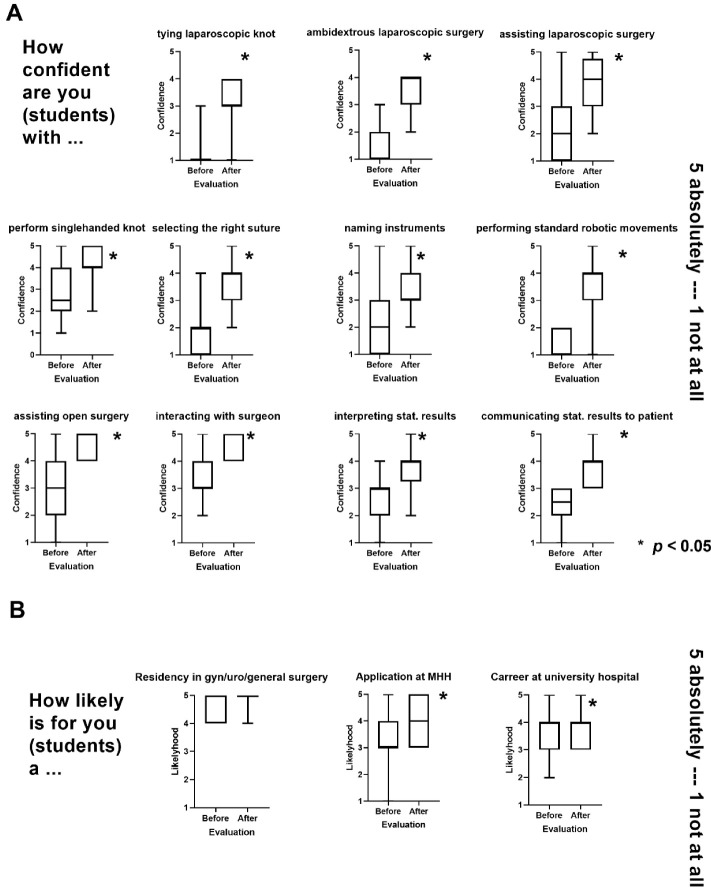
(**A**) Evaluation of the participants’ confidence in different dimensions that should be improved by SOCIUS mentoring. (**B**) Future career plans of students. Boxplots of the values before and after participation in the SOCIUS program are shown. Scoring was on a scale from 1 (lowest agreement) to 5 (highest agreement). MHH = Hannover Medical School; gyn = gynecological, uro = urological.

After participation in the SOCIUS mentoring program, students reported a significant increase in confidence related to surgical techniques, safety, and demeanor in the surgical environment, and handling of statistical results (see Figure 2). There was a particularly great increase in confidence related to laparoscopic surgical techniques. Mean confidence scores for performing a laparoscopically performed knot increased from 1.21 to 3.13 on a five-point Likert scale to the question “How confident are you with?” (1 “not at all”–5 “completely”). Particularly high confidence values were shown for the domains “interaction with the surgeon” and “assisting open surgical operations” with mean values of 4.56 and 4.63, respectively.

Additionally, after participation in the curriculum students reported higher interest in a career at a university hospital in general and our clinic specifically (Figure 2B).

## 4. Discussion

We describe the first implementation of an extracurricular program that combines individual mentoring with surgical and soft skills training. We demonstrated a high interest and need for such dedicated programs and that participation in this program significantly improves confidence towards the trained skills and leads to a specification of career goals.

Individualized support for students appeared to be essential to address the shortage of young surgeons. According to a survey by Brundage et al. at Stanford University, 44% of the students surveyed were considering a career in surgery at the beginning of their studies. After their fourth year only 27% of the students were still interested in a surgical career [14]. Peel et al. analyzed 122 articles on factors that influence students’ choice for a surgical career. The authors identified gender and surgical training as two of the three core factors determining surgical career choice [15]. Additionally, studies showed that advanced students felt insecure before surgical internships due to lack of knowledge, lack of surgical skills, fear of bad experiences, and high expectations [16]. On the other hand, high satisfaction with internships and active integration before and during surgeries stand out as positively associated factors [17]. These results clearly show our responsibility as teachers and indicated the need of early training and mentoring to accompany and support students on their way to a surgical career. Mentoring programs have been shown in several medical disciplines to have very positive effects on student satisfaction and development [4,7,9,10,11,13]. University hospitals in particular depend on motivated and ambitious young physicians to participate in clinical and translational research. The results of previous studies on what motivates students to pursue surgical careers suggest that early integration of students into clinical surgical work increases the likelihood of a surgical career [15]. By introducing a mentoring program tailored to students interested in surgery, we have developed a tool to motivate and prepare them for a surgical career. By the addition of a surgical skills training to the curriculum, we equip the students with the above described desired abilities to actively participate in surgical procedures at an early age. This aspect has been shown to be associated with increased satisfaction [15]. Participants of SOCIUS reported high satisfaction with the course. Additionally, the students reported increased confidence regarding surgical and soft skills as well as specification of career goals after mentoring. Their already high interest for a career in urology, gynecology, or general surgery and a career at a university hospital in general and our clinic specifically increased even more after the course.

In addition to professional qualifications, self-confident but always self-critical, situation-appropriate communication is one of the key competencies for a successful physician. Effective communication with patients can increase the effectiveness of therapeutic measures, and constructive critical discussion in the team across all hierarchical levels has positive effects on the error culture in the hospital [18,19]. Finally, students and young doctors also benefit personally from a professional communication style, since all communication in the hospital is a direct component of ongoing self-marketing, which, in addition to professional qualifications, has a significant influence on one’s career. This is why negotiation and communication training of students play a significant role in SOCIUS.

The confidence of SOCIUS participants increased in all areas covered by the program. In particular, the most significant increase was demonstrated for the self-assessment of operative skills, which can be explained by the students’ limited prior knowledge, especially in laparoscopic surgery. Although the subjective character of the students’ assessment limits the interpretation, it could be shown that methods for the self-assessment of students correlate with the objectifiable results and are reliable survey methods [10,13,20]. The high satisfaction and recommendation rates of participants indicate a high level of student interest in specific, individualized courses, even when offered in addition to normal curricular studies.

Statistical literacy is essential for physicians to critically and realistically evaluate scientific studies as well as their own clinical outcomes in their later medical careers. In the present study, participants reported the lowest confidence scores for statistical literacy. Regular statistical exercises with real clinical questions over a longer period of time could be suitable to anchor the basic principles more deeply. Furthermore, we plan the standardization of the candidate selection process using predefined score cards and a specific training for the mentors.

Due to the modular nature of SOCIUS, adaptation or expansion of the concept to almost any surgical clinic in any surgical subdiscipline is easily possible. Furthermore, the high application numbers indicated the high level of student interest in a highly specific program. Low absenteeism and the willingness of participants to attend courses and surgical training sessions on evenings and weekends underscore the need on the one hand and the high motivation of students on the other. Whether the participants of the SOCIUS program will actually decide to pursue a surgical career in about 3–5 years and to what extent SOCIUS can thus also be considered a success for the organizing hospitals cannot be conclusively assessed today. However, the interest in an academic career and for an application at our university increased after the curriculum and that appears to be an indication for the sustainability of this project.

Recruiting and motivating young students will certainly not completely solve the long-term shortage of physicians. The dissatisfaction of physicians working in surgery with their working environment and with often poorly structured postgraduate training must be brought into the focus of politics, professional societies and professional associations, irrespective of the recruitment of new physicians [21].

Mentoring programs should be one of the basic building blocks of active and strategic human resources management at every hospital. Interdisciplinary programs, such as the SOCIUS program, represent a valuable tool for bundling the opportunities and competencies of individual departments and for generating enthusiasm among students for a surgical career.

## Figures and Tables

**Table 1:** Characteristics of the participating students.

Characteristics	Median (Range)
Age (years)	23 (20–29)
Time spent studying (hours per week)	56.0 (15–80)
	N (%)
Female/Male	16 (80%)/4 (20%)
Current semester	N = 17
5/6	3 (17.6%)
7/8	4 (23.5%)
9/10	8 (47.0%)
11	2 (11.8%)
Previous experience in surgical training	10 (58.8%)
Already started a doctor thesis	13 (76.5%)

## Data Availability

Data are available on request from the authors.

## Supplementary Materials

The following supporting information can be downloaded at: https://www.mdpi.com/article/10.3390/medsci10030035/s1, Figure S1: Improvement of the mean total time of different laparoscopic exercises by laparosckopic training.
