# Supplementary material for: SOCIUS Mentoring—A Novel Course to Encourage Students for a Career as Surgical Oncologists
Source: Med Sci (Basel). 2022 Jun 24;10(3):35. doi: 10.3390/medsci10030035 (PMC9326547; doi:10.3390/medsci10030035)
Supplement: Supplementary file 1 [file medsci-10-00035-s001.zip › medsci-1716239-supplementary.pdf]

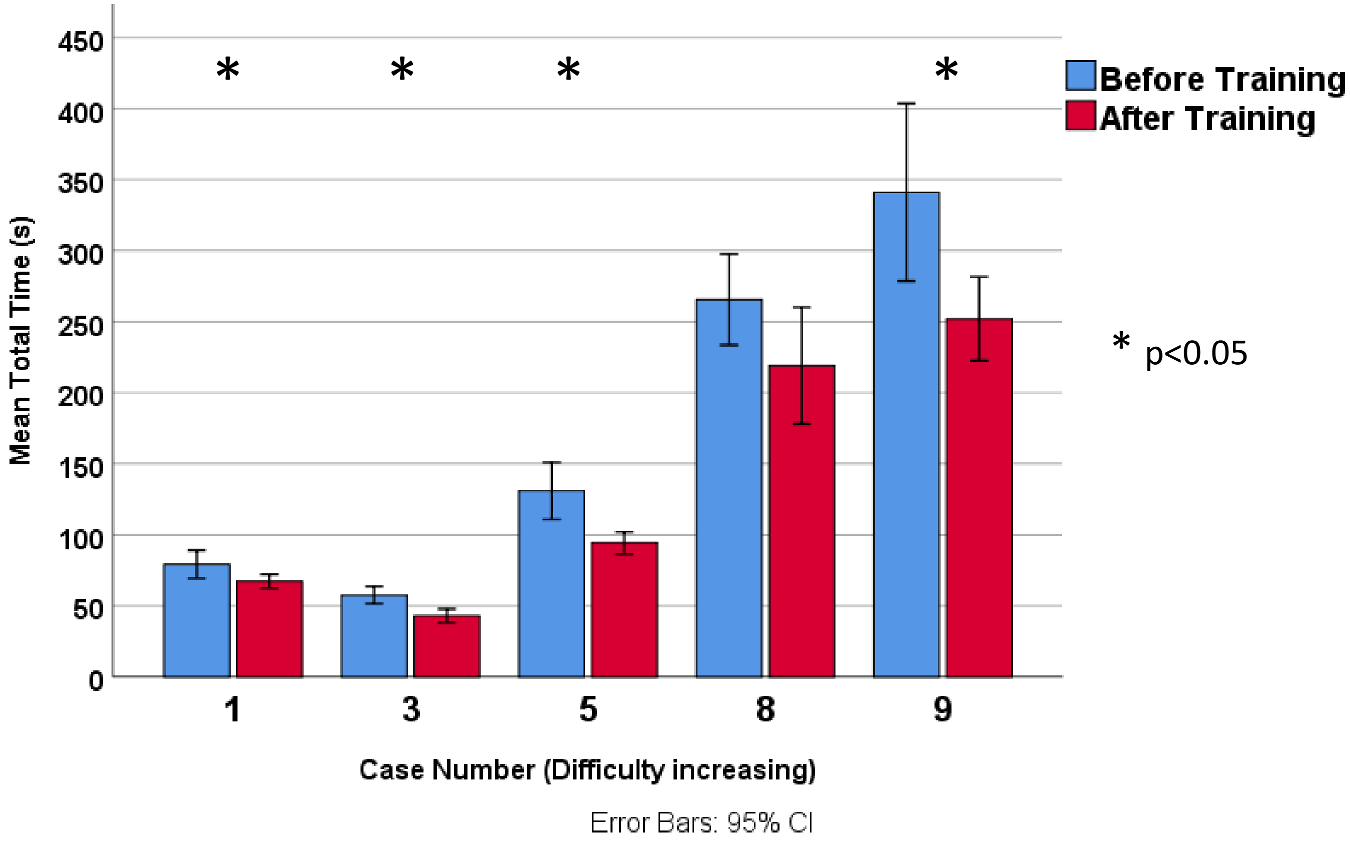

Number	Case name (Lap Mentor III, Basic Skills Module)
1	Camera Manipulation
3	Eye-Hand-Coordination
5	Clipping and Grasping
8	Electrocautery
9	Translocation of objects
